# Realism of procedural task trainers in a pediatric emergency medicine procedures course

**Published:** 2015-04-20

**Authors:** Allan Shefrin, Afshin Khazei, Adam Cheng

**Affiliations:** 1 Division of Emergency Medicine, Children’s Hospital of Eastern Ontario, University of Ottawa; 2 Department of Emergency Medicine, Vancouver General Hospital, University of British Columbia; 3 Department of Pediatrics, Alberta Children’s Hospital, University of Calgary

## Abstract

**Background:**

Pediatric emergency medicine (PEM) physicians have minimal experience in life saving procedures and have turned to task trainers to learn these skills. Realism of these models is an important consideration that has received little study.

**Method:**

PEM physicians and trainees participated in a day long procedural training course that utilized commercially available and homemade task trainers to teach pericardiocentesis, chest tube insertion, cricothyroidotomy and central line insertion. Participants rated the realism of the task trainers as part of a post-course survey.

**Results:**

The homemade task trainers received variable realism ratings, with 91% of participants rating the pork rib chest tube model as realistic, 82% rating the gelatin pericardiocentesis mold as realistic and 36% rating the ventilator tubing cricothyroidotomy model as realistic. Commercial trainers also received variable ratings, with 45% rating the chest drain and pericardiocentesis simulator as realistic, 74% rating the crichotracheotomy trainer as realistic and 80% rating the central line insertion trainer as realistic.

**Conclusions:**

Task training models utilized in our course received variable realism ratings. When deciding what type of task trainer to use future courses should carefully consider the desired aspect of realism, and how it aligns with the procedural skill, balanced with cost considerations.

## Introduction

Pediatric Emergency Medicine (PEM) physicians must be able to perform life-saving procedures such as chest tube insertion, pericardiocentesis, cricothyroidotomy and femoral venous line insertion.^1^–^4^ Unfortunately, PEM trainees reported little opportunity to perform these procedures during training.^5^,^6^ Work hour restrictions, lower patient to house-staff ratios and loss of learning opportunities to other professionals are possible contributing factors to these limited experiences.^7^–^8^

The incorporation of task trainers into curricula is helping fill training gaps. Procedures including airway management, chest tube insertion, lumbar puncture and central line insertion have been taught using them.^9^–^14^ While multiple articles discuss procedural training boot camps, their focus is on the effectiveness of the course itself, not on the individual task trainers. No work published to date has described a curriculum using a mix of homemade and commercially available task trainers while also assessing the relative value of the different task trainers.

Degree of simulator realism has the potential to enhance the educational experience. In a recent meta-analysis of the pediatric simulation education literature, Cheng et al. demonstrated that technology-enhanced simulation had a favourable effect on knowledge, time required to perform a task, behaviours and skills for pediatrics.^15^ Additionally, high realism simulators showed small to moderate benefits over low realism simulators.

But how is realism defined in medical simulation? In the past the term fidelity has often been used interchangeably with realism. The term *fidelity* is often understood to describe the mannequin only, whereas *realism* encompasses the whole simulation experience. Rudolph et al. highlight three ways in which humans consider reality in simulation: 1) physical, 2) conceptual, and 3) emotional and experiential realism.^16^ For procedural simulation, high physical realism, or highly realistic physical properties of the task trainer, is desirable to develop kinesthetic awareness and muscle memory.^16^ There is little agreement as to what defines a high or low realism simulator and there is movement toward defining realism to include not only the physical characteristics of a task trainer but also the learner’s experience of the simulation exercise.^17^ Little is known about what learners prefer when considering realism in task training or how realism correlates with effectiveness.

We designed a PEM procedural skills training course utilizing skills stations with homemade and commercially available trainers with expert instruction combined with web-based learning materials. Our study included the participant’s rating of the realism of the task trainers. Our goal was to determine the perceived realism for the task trainers, and to see if our course improved comfort, willingness and reported time to perform these procedures amongst the participants.

## Methods

The British Columbia Children’s Hospital Behavioural Research Ethics Board approved this pilot study of task training for PEM physicians and trainees.

### Participants

Participants were recruited from the Emergency Department staff and PEM fellowship program at British Columbia Children’s Hospital. All staff physicians and fellows were eligible for inclusion. Individuals were excluded if they received training in any of the procedures outside of regular medical school curricula, residency and fellowship training and Advanced Trauma Life Support (ATLS^®^) courses.

### Study Design

Subjects participated in a daylong procedural skills training program focusing on skills training for chest tube insertion, pericardiocentesis, cricothyroidotomy and central line insertion. They were asked to review four web-based modules prior to the course as in a “flipped” classroom model. Instructors were PEM, pediatric intensive care, pediatric cardiology and adult emergency medicine attending physicians with a minimum of 10 years clinical experience, prior experience teaching using simulation-based methodologies and procedural expertise for the assigned skill station.

During the course, groups of three participants spent 45 minutes at each skills station where an expert was present to demonstrate the procedure and provide feedback. One commercially available and one homemade task trainer were available for all procedures except for central line insertion (commercial trainer only). Homemade task trainers describe a trainer that could be made from readily available or store purchased items such as gelatin, rolls of tape, balloons. Appendix 1 shows the task trainers used for the course with their approximate cost. Ultrasound guidance was used for pericardiocentesis and central line insertion.

### Outcome measures and data analysis

Prior to the course, participants were asked to complete a pre-course survey that assessed their baseline experience, comfort, willingness and time to perform the four procedures. After the course, a post-course survey was completed reassessing these variables and participants rated how “real” each of the homemade and commercially available task trainers appeared to them. Survey responses were gathered using 5-point Likert scales, which were condensed to 3-point scales for comparison due to the small sample size. Mean scores were calculated for survey responses and pre and post averages were compared. Means, standard deviations and 95% confidence intervals (95% CI) were calculating using Microsoft Excel 2011 for Mac (Microsoft Corporation, Redmond, WA).

## Results

A convenience sample of 12 participants enrolled in the course included six attending physicians and six fellows. Fellows were in their first or second year of PEM training. Attending physicians varied between one and 20 years of clinical experience. Pre and post course surveys had a 100% response rate. Previous procedural skills experience of our participants is reported in Table 1.

Participants’ perception of realism of homemade and commercially available task trainers was variable. The homemade pork rib chest tube model and gelatin pericardiocentesis model were rated realistic by a large majority of respondents (91% and 82% respectively), while only 45% of respondents rated the commercially available chest drain and pericardiocentesis trainer as realistic. Conversely, 36% rated the homemade ventilator tubing cricothyroidotomy model as realistic, while 73% rated the commercially available cricotracheotomy trainer as realistic. 80% of respondents rated the commercially available femoral line task trainer system as realistic.

Participants rated all aspects of the course highly. Comfort and willingness to insert chest tubes showed significant improvement. All other variables trended toward improvement (Table 2).

## Discussion

Our cohort of PEM physicians and trainees improved their skills by participating in a daylong procedural training course utilizing homemade and commercially available task trainers. These models exhibited variable realism as perceived by the participant, with some homemade models being perceived as highly realistic. Considering the three domains of realism (physical, conceptual and emotional) in medical simulation defined by Rudolph and colleagues, we sought to use highly physically realistic task trainers.^16^ The course was not structured to target conceptual and emotional realism. Learners had variable perceptions when asked to rate the degree of realism for each task trainer. In two instances, the homemade models were more realistic (chest tube insertion – animal tissue; pericardiocentesis – gelatin mold), while in another the commercially available model was more realistic (cricothyroidotomy). It is unclear whether higher realism task trainers led to increased learning as our study was not designed to do this; we used both high and low realism task trainers at some stations. A future area of investigation would be to determine if higher realism trainers lead to improvements in comfort, willingness to perform and perceived time to complete a specific procedure. Perhaps more importantly, it is interesting to assess the effect of higher realism models on performance using validated scoring tools or checklists for these procedural skills.

Taking the position that the concept of *fidelity* is flawed, Hamstra and colleagues propose that educators focus on the underlying principles for effective learning.^17^ Functional task alignment emphasizes the importance of the task and the need for an active and intentional process to determine the needed alignment. In this construct, emphasis is shifted away from physical resemblance provided there is appropriate correspondence between the functional aspects of the simulator and the applied context.^17^ Perhaps this explains why the gelatin pericardiocentesis mold was rated as realistic by more participants than the commercially available trainer. This homemade model did not resemble a chest but allowed for ultrasound visualization of the pericardial effusion and draining of it. The commercially available model exhibited only superficial realism as it looked very much like a chest but did not allow visualization of the specific task.

Cost does not equal realism. The commercially available trainers were considerably more expensive than homemade ones. In this study, more participants viewed as realistic the $20 pork rib model for chest tube insertion and the $70 gelatin pericardiocentesis mould compared to the commercially available counterparts. Homemade models require time and energy to collect supplies and assemble the model whereas commercial models require research into the model that best fits the need and budget. All commercially available task trainers require ongoing maintenance and/or replacement parts. That lower cost simulators can deliver a realistic learning experience is promising news for lower budget programs, especially those in developing countries.

This study has several limitations. Only one homemade and one commercially available task trainer in each domain were tested, except for central line insertion where no homemade model was used. Perhaps other commercially available models or other recipes for homemade trainers would be more realistic. We did not measure participant compliance with viewing the web-based material, and thus this may have influenced our results. Longer term retention of skills also needs to be assessed, especially when considering the realism, or lack thereof, of certain task trainers. There were also very few tasks in this study rendering a correlation between ratings of realism and amount learned impossible. More and larger studies are required to assess the best environments for these or other task trainers.

## Conclusion

This study of pediatric emergency physicians and trainees showed that inexpensive homemade task trainers and commercially available task trainers can be effective. Whether there is a relationship between realism of the task trainer and the amount learned requires further study. Educators should select task trainers and teaching environments with functional task alignment keeping in mind the needs of the learner and the costs of the model.

## Figures and Tables

**Table 1:** Number of procedures performed in participant’s career

	Reported procedures performed n the participant’s career *n* (%)			
Procedure	0	1–2	2–4	≥5
Chest tube insertion	1(8)	4(33)	2(16)	5(46)
Pericardiocentesis	11(92)	1(8)	0(0)	0(0)
Cricothyroidotomy	12(100)	0(0)	0(0)	0(0)
Central line insertion	1(8)	3(25)	4(33)	4(33)

**Table 2:** Pre and post course scores for comfort, willingness to perform and reported time to perform each procedure

	Pre-course score (95% CI)	Post-course score (95% CI)
**Chest Tube Insertion**		
Comfort (/5)*	3.25 (2.61–3.89)	4.55 (4.23–4.85)
Willingness to perform (/5)*	3.50 (3.21–3.79)	3.91 (3.81–4.01)
Time (min)	5.40 (3.98–6.88)	3.88 (2.65–5.05)

**Pericardiocentesis**		
Comfort (/5)	2.00 (1.32–2.68)	2.82 (2.24–3.40)
Willingness to perform (/5)	2.67 (2.23–3.11)	2.91 (2.50–3.32)
Time (min)	4.38 (2.65–6.11)	2.95 (1.50–4.40)

**Cricothyroidotomy**		
Comfort (/5)	2.33 (1.72–2.94)	3.27 (2.67–3.87)
Willingness to perform (/5)	2.83 (2.42–3.24)	3.18 (2.94–3.42)
Time (min)	4.10 (2.20–6.00)	3.18 (1.85–4.51)

**Central Line Insertion**		
Comfort (/5)	3.42 (2.60–4.24)	4.50 (4.17–4.83)
Willingness to perform (/5)	3.08 (2.52–3.64)	3.70 (3.40–4.00)
Time (min)	7.05 (5.60–8.50)	4.75 (3.05–6.45)

* denotes statistically significant result

**Appendix 1:** Description and Cost of Homemade and Commercially Available Task Trainers

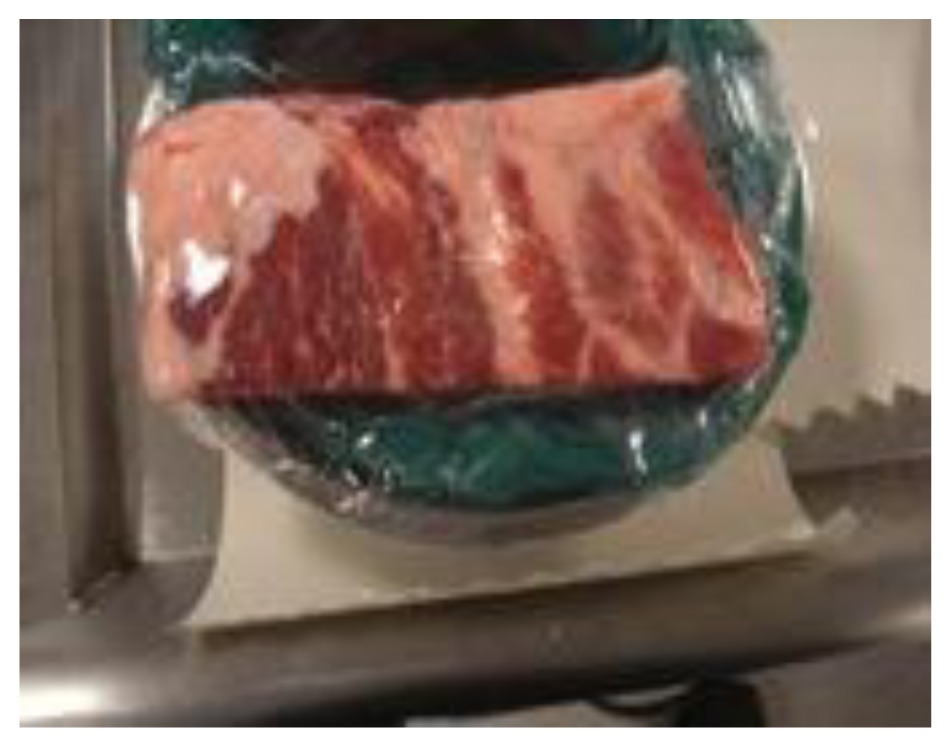	Homemade chest tube insertion trainer Approximate cost: $20/model
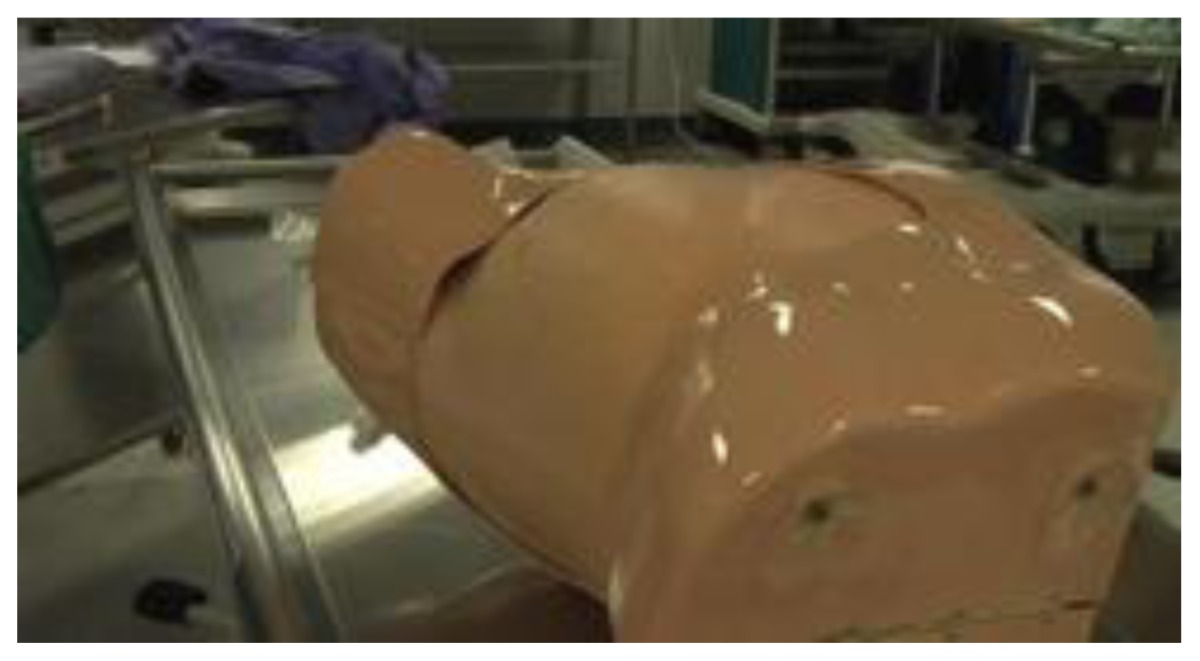	Commercially available chest tube and pericardiocentesis trainer* Approximate cost: $1800/model
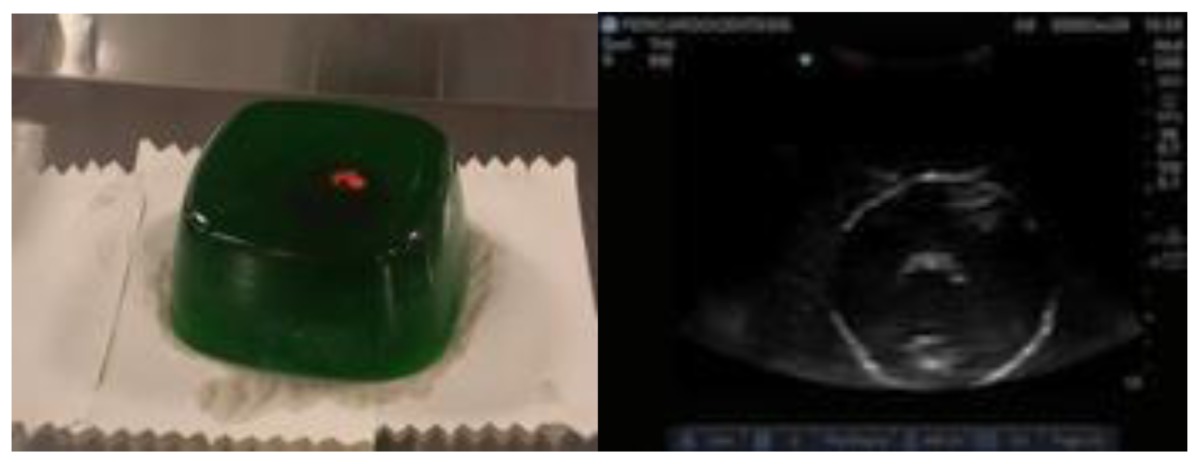	Ultrasound capable homemade pericardiocentesis model+ Approximate cost: $70/model
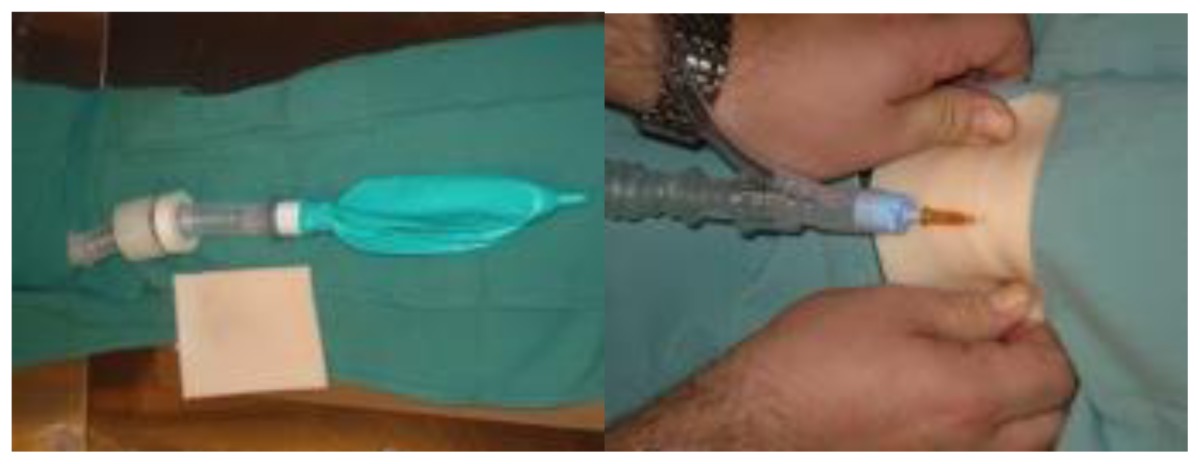	Homemade cricothyroidotomy trainer# Approximate cost: $10/model Commercial Cricotracheotomy Trainer (image not shown) ++ Approximate cost: $1200/model
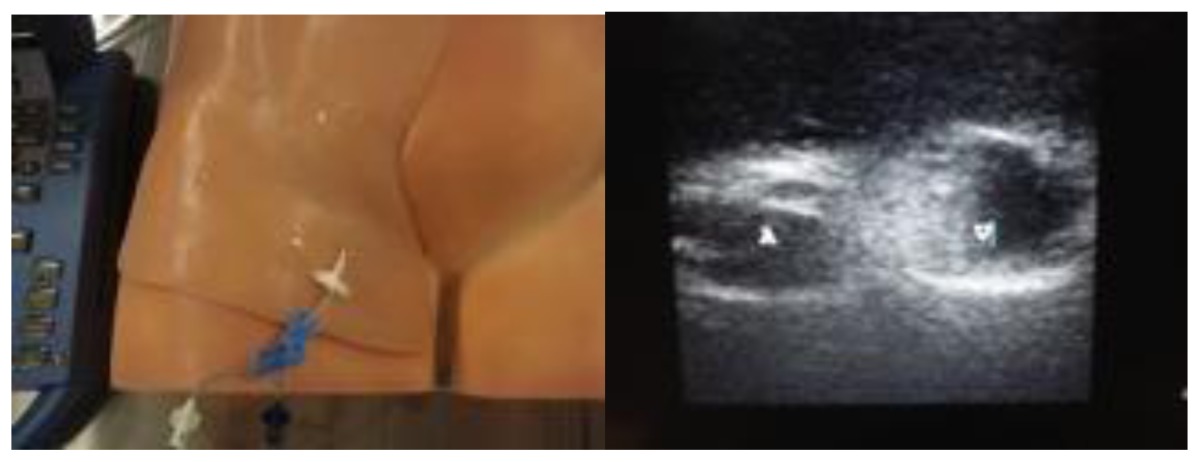	Ultrasound capable commercially available vascular access trainer$ Approximate cost: $1500/model

* CDT100 Chest Drain Insertion Simulator (Pharmabotics Ltd., Hampshire, United Kingdom);

+ Gelatin with balloon mold for pericardiocentesis (personal communication with Dr. Herb Zerth);

# Homemade cricothyroidotomy task trainer (Varaday SS, Yentis SM, Clarke S. A homemade model for training in cricothyrotomy. Anaesthesia, 2004, 59(10):1012–15.);

++ Pharmabotics Ltd, Hampshire, UK;

$ FemoraLineMan System (Simulab Corp., Seattle, WA) with femoral line in place
